# COVID-19 and Psychiatric Admissions: A Comparative Study of Pre-pandemic and Post-pandemic Psychosis Admissions in a South Florida Emergency Department

**DOI:** 10.7759/cureus.40989

**Published:** 2023-06-26

**Authors:** Clinton A Ross, Sam Kara, Gerardo F Ferrer

**Affiliations:** 1 Psychiatry, Larkin Community Hospital, Miami, USA; 2 Neurology, Larkin Community Hospital, Miami, USA

**Keywords:** covid-19, psychiatry, social issues, community psychiatry, psychosis, epidemiology and biostatistics

## Abstract

We noticed a subjective increase in psychosis admissions within our emergency department (ED) with the onset of the coronavirus disease 2019 (COVID-19) pandemic. This study aimed to identify trends concerning admissions due to psychosis in the ED before and after the beginning of the COVID-19 pandemic.

We analyzed 508 psychiatric admissions through the ED from October 2019 to October 2020, of which 367 cases of psychosis were identified. Statistical analysis was performed using T-tests and Pearson's correlation coefficient.

T-testing showed mean psychosis admissions during the pandemic (March 2020 to July 2020) to be greater than admissions occurring during the pre-pandemic period (October 2019 to February 2020) (p = 0.04). Pearson's correlation coefficient identified the relationships between COVID-19 admissions and psychosis admissions during this time as positive (r = 0.5) but did not reach statistical significance (p = 0.06). Therefore, within our time frame, we did see a noted increase in psychosis by 22.9% during the pandemic compared to pre-pandemic times.

Current research remains conflicted concerning psychiatric ED admissions during COVID-19, with some stating an increase and others finding a decrease. Our data showed a significant statistical increase in the mean number of psychosis cases when comparing pre-pandemic and pandemic admissions. These findings help add pertinent data to understand how psychosis admissions trended before and during the beginning of the COVID-19 pandemic, specifically in South Miami, Florida. It also provides a foundation for future studies by providing data points concerning mental illness within the vulnerable population of patients served in our community.

## Introduction

Severe acute respiratory syndrome coronavirus 2 (SARS-CoV-2) is the contagious virus known for causing the coronavirus disease 2019 (COVID-19) pandemic. The World Health Organization (WHO) states that the original outbreak began in Wuhan, China, in December 2019. By March 11, 2020, COVID-19 became a global, ongoing pandemic [1]. Methods employed to decrease contact with the contagion, such as lockdowns and social distancing, effectively increased isolation, which led to exacerbating psychiatric illnesses [2,3]. Some individuals reported feelings of alienation, entrapment, and loneliness, leading to increased suicidal tendencies [4]. Others identified the increased stress of the COVID-19 pandemic, in general, to have affected their mental illness. Historically, it is known that psychiatric patients will undergo worsening of their psychiatric symptoms if they are not compliant with medications [5]. As such, during the pandemic, many patients with non-communicable diseases, such as psychiatric illnesses, also had disruption of treatment due to issues with accessing medications [6]. Lack of access to mental health care, whether due to lack of insurance or available resources such as medications or providers, presented another factor for exacerbating psychiatric illness [5,7]. Along with the aforementioned, reports also emerged that increased economic stressors, substance abuse, domestic violence, and exposure to stories of hopelessness via media outlets led to the decompensation of psychiatric illnesses and increased involuntary admissions [4,8,9].

Overall, a well-documented history of epidemics exacerbating psychiatric illnesses exists. In the United States, suicide rates increased during the Spanish flu epidemic in 1918-1919 [10]. An increase in post-traumatic stress disorder (PTSD), depression, and suicide deaths also occurred during the severe acute respiratory syndrome (SARS) epidemic in 2003 [10,11]. However, results appear conflicted when examining the different trends in psychiatric admission rates at various emergency departments (EDs) worldwide concerning the onset of COVID-19. For example, the trend of psychiatric illness admissions increased in Spain's population but decreased in Switzerland's. Different psychiatric illnesses also appeared to have different trends for different populations during the pandemic. For example, in Spain, anxiety disorders presented less often than usual, while autism, substance use, and dementia presented more often during this time [12]. Although the Swiss reported decreased admissions for psychiatric illness during the COVID-19 pandemic, it also highlighted that psychiatric patients were significantly more likely to be involuntarily hospitalized [13]. Concerning the US population, there was reported an increase in general psychiatric symptoms during COVID-19. An increase in depressive symptoms was reported from a study assessing depression symptoms in US adults both before and during the COVID-19 pandemic. The study utilized the Patient Health Questionnaire-9 (PHQ-9) to show an increase in depressive symptoms after the pandemic onset. When comparing the prevalence of depression symptoms during pre-COVID and COVID-19, they tripled across the nation in men and women aged 18 to 60+ years old. Before the pandemic, depression rates were 8.5% and rose to 27.8% during the pandemic. Of note, those who reported having more resources, being married or living with a partner, having higher income, having less stress, and having at least 5000$ in savings appeared to report fewer depressive symptoms. Women reported more depressive symptoms overall during COVID-19 (before 277 women (10.1%) vs. 181 men (6.9%); during 233 women (33.3%) vs. 149 men (21.9%)). Regarding ethnicity, non-Hispanic Asians (eight participants (23.1%) vs. 26 participants (4.4%)) reported the most depressive symptoms. Age did not appear to correlate with reported depressive symptoms. Depression symptom prevalence was 1.5 times higher for mild depression symptom scores, 2.6-fold higher for moderate depression symptom scores, 3.7-fold higher for moderately severe depression symptom scores, and 7.5-fold higher for severe depression symptom scores categories [14]. Beyond depressive symptoms, the US Census Bureau also found that the prevalence of depression in the general population increased slightly right after the pandemic was announced. Adults were also three times as likely to screen positive for depression, anxiety, or both diagnoses during that time [15]. Concerning the global prevalence of depression, a meta-analysis shows that the worldwide prevalence of depression increased sevenfold from 3.44% to 25% [16].

In the US, there have been case reports of COVID-19 and psychosis occurring together and also patients presenting with new onset psychosis possibly due to COVID-19 itself [17,18]. A study conducted in New York City (NYC) showed that psychosis diagnoses increased among all patients regardless of COVID-19 status; however, they added that there was also a 242.5% increase in new-onset psychosis among COVID-19-negative patients. However, they also noted that the data were not representative of the US population but only of the population of certain boroughs in NYC. The authors further stated that although a possible relationship between COVID-19 infections, hospitalizations, and psychiatric diagnoses appears to be present, no estimate for causality was attempted due to requiring further adjustments for potential confounding factors [19].

Within our population, we admitted a few complaints of altered mental status, aggressive behavior, and suicidal behavior at our facility during our study. Concerning diagnoses, we also admitted depression, bipolar, dementia, alcohol withdrawal, anxiety, and disruptive mood dysregulation. Subjectively the trend did not appear to change for those mentioned above. However, in looking at diagnoses for psychosis, we subjectively noticed psychosis admissions to be substantially elevated after the COVID-19 pandemic onset.

Unfortunately, data giving insight into the trends of psychiatric ED admissions during COVID are not available for the US, in general, South Miami, or any other region of Florida. Therefore, this study aimed to identify trends concerning admissions of psychosis in the ED before and after the onset of the COVID-19 pandemic. We also aimed to investigate the causal relationship between COVID-19 and psychosis in our population. Overall, the goal is to add to the current body of existing data to understand better how COVID-19 impacted ED admission rates for psychiatric illness in Florida and across the United States.

## Materials and methods

Before initiation, the study was submitted to and approved by the Larkin Community Hospital Institutional Review Board under DRAA file code R-0423DS/CR. All patient information was de-identified, and informed consent was obtained via written documentation before the data were marked for inclusion in the study. The period selected for our research coincided with dates before and after federal and state-mandated restrictions came into effect in South Miami, Florida. To identify our patient population, we created a report utilizing data from our electronic medical records according to specific search criteria. First, we included those who provided consent and those who presented with a psychiatric diagnosis to our ED from October 2019 to October 2020. For those aged 17 years and younger, consent was provided via form upon admission. Any patient not giving consent does not appear when we search for them in our database. The International Classification of Diseases (ICD) coding was used to determine the psychiatric diagnosis. The ICD codes included are as follows: all diagnostic codes in between F20 and F29 (psychotic disorders) as well as F30-39 (affective disorders). Coding for COVID was as follows: B34.2 coronavirus infection, unspecified; B97.29 other coronavirus; U07.1 COVID-19; U07.2 COVID-19; J12.81 pneumonia due to SARS-associated coronavirus; positive laboratory test for 9088 - SARS coronavirus 2 and related RNA.

Patients were identified as having COVID-19 with a positive result via molecular lab test (polymerase chain reaction/nucleic acid amplification test), and patients were identified as having an exacerbation of psychiatric symptoms by a psychiatrist doing a complete psychiatric evaluation in the ED. Of note, all patients received two psychiatric assessments. An initial psychiatric evaluation was completed by a first, second, third, or fourth-year psychiatry resident, with the subsequent evaluation conducted by a board-certified psychiatrist. No scales, such as the positive and negative syndrome scale (PANSS), were used in the ED setting concerning objective psychiatric symptoms; the diagnosis was based on clinical criteria outlined in the Diagnostic and Statistical Manual of Mental Disorders, 5th Edition (DSM-V).

The initial data were organized and tabulated by diagnosis, number of cases per month, and case totals for each corresponding month and the entire 12-month period. These data points were then put together in a graphical format to assess if any potential trends were present within our data.

The data were further isolated into two distinct groups concerning our primary endpoint. Those with psychotic symptoms were grouped together, which included diagnoses as follows: schizophrenia, other schizophrenia, schizophrenia not due to a substance or known physiological condition, brief psychotic disorder, delusional disorders, and unspecified psychosis. The other group consisted of patients diagnosed with new-onset COVID-19 and a co-existing psychosis exacerbation, whether new onset or previously diagnosed.

All patients admitted to the psychiatric unit undergo urine drug screening (UDS) in the ED. Patients must also be medically stable before being transferred to the psychiatric unit. Therefore, no patients included in the study were in acute intoxication or withdrawal states. Any diagnosis of substance or medically induced psychosis was left out of this study to reduce confounding and to focus solely on psychiatric illness. Substance use diagnostic codes were not utilized either.

Statistical analysis was then performed on the data obtained five months before the pandemic (October 2019 to February 2020) and five months after the onset of the pandemic (March 2020 to July 2020) to establish the presence of statistical significance. Two-tailed and one-tailed T-tests were utilized to verify if there was a significant increase in psychosis during our designated time. Correlation coefficients were calculated to identify if having a diagnosis of COVID contributed to having psychosis or not as well. Statistical analysis was performed using StatPlus:mac, version v8 by AnalystSoft Inc. (Alexandria, VA). Contribution percentages were also calculated during our designated pre-pandemic and pandemic times to quantify trends.

Demographics, including sex, ethnicity, age, length of service, and the average length of service for our entire population, were identified. Concerning sex and ethnicity, the total values for each of the two groups we tabulated, and percentages were assessed to understand the patient distribution. Comparisons were attempted concerning sex, ethnicity, and age concerning any noted psychiatric admission trends as appropriate. Contribution percentages were also calculated to assess if the sample population represented the local community.

Lastly, a literature review was used to identify variables thought to have been associated with COVID-19, exacerbation of psychiatric symptoms, and ED admission rates.

## Results

Table 1 represents the final results of the data inquiry performed and utilized throughout the study. We initially identified 515 data points; however, the number was reduced to 508 due to some patients being accounted for more than once. The data revealed 367 psychosis-only cases and 22 cases where psychosis and COVID-19 occurred concurrently.

**Table 1:** ED admission totals from October 2019 to October 2020 by corresponding diagnosis/chief complaint The diagnosis of each consenting patient admitted through the Emergency Department at Larkin Community Hospital between October 2019 and October 2020 was included alphabetically in the first column. Each case was then assigned a value of 1 and separated by the corresponding month they presented to the ED. The total occurrence of each case per month was then summated and denoted with a value under the corresponding month in columns 2 through 14. The final column shows how many cases were admitted to LCH ED per diagnosis across the 12 months of our study. The total row at the bottom of the table denotes the total number of cases per month regardless of the diagnosis, with the final column in the last row being the total number of cases included in the study. This value was calculated by adding all the values in column 15 together. LCH ED: Larkin Community Hospital Emergency Department; AMS: altered mental status; CHF: congestive heart failure; MVA: motor vehicle accident; PEG: percutaneous endoscopic gastrostomy; SIRS: systemic inflammatory response syndrome.

	The month of admission through LCH ED													
Admitting diagnosis	19-Oct	19-Nov	19-Dec	20-Jan	20-Feb	20-Mar	20-Apr	20-May	20-Jun	20-Jul	20-Aug	20-Sep	20-Oct	12-month case total (per diagnosis)
Abdominal distension											1			1
Abdominal pain						1								1
Acute anemia														0
Acute colitis											1		1	2
Acute kidney injury							1							1
Acute renal failure					1									1
Age-related cognitive decline										1				1
Aggressive behavior	1			1					1	1				4
Alcohol withdrawal						2						1		3
AMS				2									1	3
Anxiety								1						1
Bipolar disorder			1					1	1		2		1	6
Bizarre behavior	1	2		1	1	1							1	7
Chest pain		1												1
Complicated CHF		1												1
Complicated abscess												1		1
Complicated UTI										1		1	1	3
Constipation				1	1				1					3
Cough	1													1
COVID and psychosis								6	1	5	8		2	22
Rash				1										1
Disruptive mood dysregulation			1											1
Dysphagia					1									1
Flu-like symptoms				1										1
Foreign body ingestion	1													1
Fracture of left humerus						2								2
Headache		1												1
Hematemesis				1										1
Hip pain		3												3
Homicidal ideation	1													1
Hypertensive crisis		1												1
Hypertensive emergency				1										1
Hypoglycemia				1										1
Hyponatremia		2			1					1		1		5
Hypotension										1				1
Hyperkalemia									1					1
Hypothyroidism											1			1
Infection of the surgical site				1										1
Intractable vomiting										1				1
Jaundice							1							1
Left leg pain		1		1										2
Major depressive disorder		1	1					2	1	1	1	2		9
Metabolic encephalopathy		1			2						1			4
MVA				1										1
Neck pain													1	1
Not feeling well					1									1
Not taking meds						1								1
PEG malfunction					1								2	3
Pneumonia						1	1	1	2		1			6
Possible overdose										1				1
Psych problem	1													1
Psychosis	17	23	20	24	6	17	24	35	40	26	46	39	50	367
Rapid atrial fibrillation											1			1
Respiratory distress				1										1
Rhabdomyolysis	1					1	1				1	1		5
Seizure disorder	1							1	1		2	1		6
Shortness of breath			1											1
SIRS														1
Suicidal ideation			1					1				1		3
Swollen extremities											1			1
Syncope												1		1
Weakness													1	1
Monthly case totals (regardless of diagnoses)	26	37	25	38	15	26	28	48	49	39	67	49	61	508 - Total of all cases in the study
	19-Oct	19-Nov	19-Dec	20-Jan	20-Feb	20-Mar	20-Apr	20-May	20-Jun	20-Jul	20-Aug	20-Sep	20-Oct	
	The month of admission through LCH ED													

**Figure 1:**
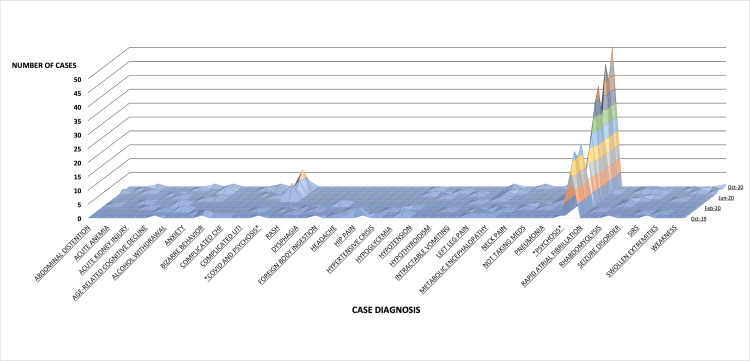
ED admission totals from October 2019 to October 2020 by corresponding diagnosis or chief complaint The x-scale corresponds to the first column in Table 1. Of note, not all diagnostic labels fit on the x-scale; however, all data from Table 1 are reflected in Figure 1. Asterisks were placed next to the diagnoses of both COVID and psychosis presenting in tandem and psychosis presenting by itself separately. The y-scale denotes the cases of each given diagnosis per corresponding month designated on the z-scale. The month list is also truncated due to scaling. CHF: congestive heart failure; SIRS: systemic inflammatory response syndrome.

**Figure 2:**
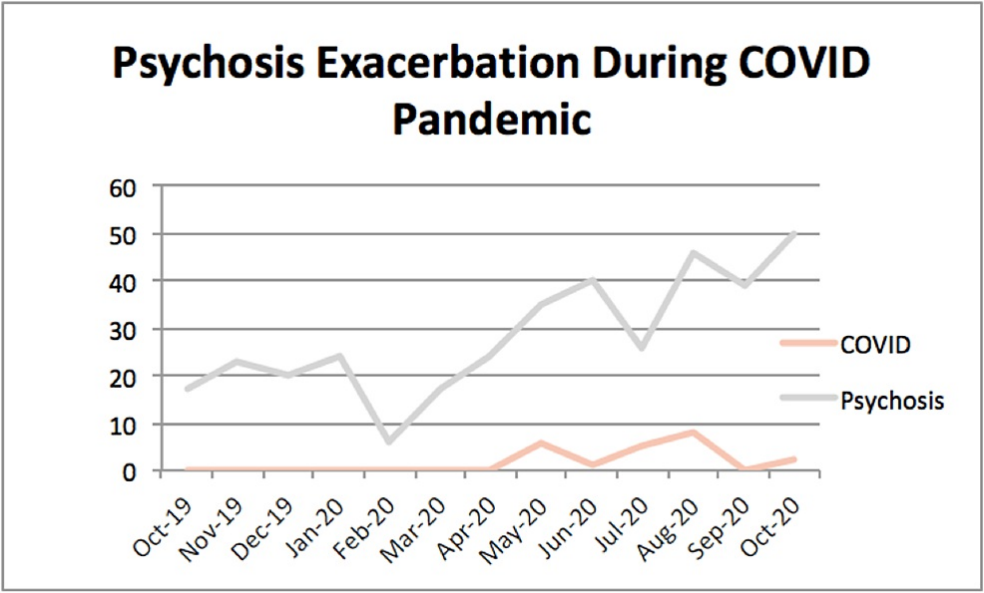
Psychosis exacerbation during the COVID pandemic

Figure 1 represents Table 1 in graphical format. There appeared to be an increase in psychosis admissions while cases of co-occurring COVID-19 and psychosis began to appear. The psychosis and COVID-19 admissions were then isolated and plotted on a separate line graph to better view this trend (Figure 2).

Table 2 depicts how many cases were presented within each given month during the study period; co-occurring COVID and psychosis contribution percentage is also shown. As previously stated, the pandemic was announced by the WHO on March 11, 2020. Before the pandemic (10/2019 to 2/2020), there were 90 cases of psychosis (38.8% of 232 total) and 0 cases of COVID-19. After the pandemic began (4/2020 to 10/2020), there were 142 cases of psychosis (61.2% of 232 total) and 12 cases of psychosis that occurred while also being diagnosed with COVID-19. Overall, COVID-19 occurred in tandem with psychosis for 5.17% of our population (12 cases of psychosis with COVID-19 out of 232 total psychosis cases). From 4/2020 to 10/2020, psychosis admissions in the setting of COVID-19 contributed to as much as 17.17%, 18.18%, and 19.23% of total cases during the months of 5/2020, 6/2020, and 8/2020, respectively (Table 2).

**Table 2:** COVID-19 and psychosis monthly case counts and contribution percentages The corresponding diagnoses for the numerical values within the table are located in column 1. The numerical values in rows 2 and 3 represent the total case number present in the study for the given months in columns 2-13. The case contribution percent in row 4 was calculated by dividing the total cases of concomitant COVID and psychosis in any given month by the total number of psychosis and COVID/psychosis cases that occurred in that month. Of note, percentages are rounded in row 4. LCH ED: Larkin Community Hospital Emergency Department.

	The month of presentation to LCH ED												
Admitting diagnosis	19-Oct	19-Nov	19-Dec	20-Jan	20-Feb	20-Mar	20-Apr	20-May	20-Jun	20-Jul	20-Aug	20-Sep	20-Oct
Number of COVID-19 and psychosis cases	0	0	0	0	0	0	0	6	1	5	8	0	2
Number of psychosis cases	17	23	20	24	6	17	24	35	40	26	46	39	50
Concomitant COVID-19 and psychosis contribution percent	0%	0%	0%	0%	0%	0%	0%	17.17%	2.50%	18.18%	19.23%	0%	4%

The ethnic groups identified in the study were Hispanic and non-Hispanic Caucasian individuals. Our population consisted of mainly Hispanic males by a narrow margin, as evidenced by Tables 3-6.

**Table 3:** Psychosis and co-occurring COVID-19 case demographics - sex

	Psychosis with co-occurring COVID-19 cases		Percentage
Sex	Male	7	(7/12) 55%
	Female	5	(5/12) 45%
	Total	12	100%
	* percentages rounded*		

**Table 4:** Psychosis and co-occurring COVID-19 case demographics - ethnicity

	Psychosis with co-occurring COVID-19 cases		Percentage
Ethnicity	Caucasian non-Hispanic	2	(2/12) 17%
	Caucasian Hispanic	10	(10/12) 83%
	Total	12	100%
	* percentages rounded*		

**Table 5:** Psychosis case demographics - sex

	Psychosis cases		Percentage
Sex	Male	128	(128/232) 55%
	Female	104	(104/232) 45%
	Total	232	100%
	* percentages rounded*		

**Table 6:** Psychosis case demographics - ethnicity

	Psychosis cases		Percentage
Ethnicity	Caucasian non-Hispanic	107	(107/232) 46%
	Caucasian Hispanic	125	(125/232) 54%
	Total	232	100%
	* percentages rounded*		

The total cases in our data with both COVID and psychosis at the same time equaled 12. Regarding sex, there were seven (58%) males and five (42%) females (Table 3). Caucasian Hispanics contributed to 10 of these 12 cases (83%) versus Caucasian non-Hispanics, who represented two out of these 12 cases (17%) (Table 4).

There were 232 total cases of psychosis exacerbation within our population. Regarding sex, 128 cases were males (55%), and 104 were females (45%) (Table 5). Concerning ethnicity, 198 out of 367 were Hispanic (54%), and 169 out of 367 were non-Hispanic (46%) (Table 6).

Our age range consisted of individuals aged 12 to 97 years for those with psychosis exacerbation. For those who presented with psychosis exacerbation and a COVID-19 diagnosis, the ages ranged from 39 to 96 years old.

The length of hospital stay for psychosis-only patients varied from one to 57 days, with an average length of service of five days. For patients with psychosis in conjunction with COVID-19, the length of hospital stay ranged from three to 56 days, with an average of 17 days of service.

Mean differences in the data represented in Table 7 were of statistical significance. Upper-tailed T-testing showed mean psychosis admissions during the pandemic (March 2020 to July 2020) to be greater than admissions occurring during the pre-pandemic period (October 2019 to February 2020) (p = 0.04053) (Table 8). Lower-tailed testing and two-tailed testing showed no significance. Linear correlation was completed using the data from Table 9. Pearson’s correlation coefficient identified the relationships between COVID admissions and psychosis admissions during this time as positive (r = 0.51628); however, not statistically significant (p = 0.06329) (Table 10).

**Table 7:** Statistical analysis data pool for means report and T-test

Psychosis admissions	
Pre-pandemic	Pandemic
(Oct 2019) 17	(Mar 2020) 17
(Nov 2019) 23	(Apr 2020) 24
(Dec 2019) 20	(May 2020) 35
(Jan 2020) 24	(June 2020) 40
(Feb 2020) 6	(July 2020) 26

**Table 8:** Statistical analysis - means report and T-test results LCL: lower confidence limit; UCL: upper confidence limit.

Means report			
	Mean	95% LCL	95% UCL
Column A (1)	18	9.00329	26.99671
Column B (2)	28.4	17.06748	39.73252
Mean difference (1-2)	-10.4	-1.6178	22.4178

T-test (homoscedastic)	
Hypothesized mean difference	0
Mean difference	-10.4
Pooled variance	67.9
Test statistic	1.99558
Degrees of freedom	8

H1: Mu1 - Mu2 > 0/greater than (upper-tailed)					
t critical value (5%)	1.85955	p-value	0.04053	H1 (5%)	Accepted
H1: Mu1 - Mu2 ≠ 0/not equal (two-tailed)					
t critical value (5%)	2.306	p-value	0.08107	H1 (5%)	Rejected
H1: Mu1 - Mu2 < 0/less than (lower-tailed)					
t critical value (5%)	-1.85955	p-value	0.95947	H1 (5%)	Rejected

**Table 9:** Statistical analysis data pool for Pearson correlation coefficient (R)

	Case count	
Month of admission	Co-occurring COVID and psychosis cases (A)	Psychosis cases (B)
Oct 2019	0	17
Nov 2019	0	23
Dec 2019	0	20
Jan 2020	0	24
Feb 2020	0	6
Mar 2020	0	17
Apr 2020	0	24
May 2020	6	35
June 2020	1	40
July 2020	5	26

**Table 10:** Statistical analysis - Pearson correlation coefficient R = correlation coefficient; N = number of valid cases.

Correlation coefficients (pairwise deletion)			
	R	N	p-value
COVID/psychosis cases vs. COVID cases	0.51628	10	0.06329

## Discussion

Presented is a retrospective study of ED admissions at a community hospital in South Miami, Florida, occurring during the first wave of the COVID-19 pandemic. This is the first report to document the presentation of psychosis and subsequent hospital admissions in Florida during the COVID-19 pandemic.

Though we believed there was an increase in psychosis cases from the onset of the pandemic, COVID was not reported to be present in southern Florida at its start. The first cases of presumptive COVID-19 reported by the Florida Department of Health (FLDOH) appeared on March 1, 2020, in Florida's Hillsborough and Manatee Counties [20]. Other sources state that COVID-19 has been in Florida since mid-February [21]. We received our first cases in South Miami in May 2020 (Table 2).

March 11, 2020, marks the date for the first confirmed case of COVID-19 in Miami-Dade, while Broward County was confirming its 5th case then. Initially, we believed admissions for COVID-19 would appear at the pandemic's beginning since Miami-Dade is a tourist-heavy county; however, they began appearing during May instead. It should be noted that though COVID cases did not present at our hospital until a couple of months after the onset of the pandemic, most likely COVID was already spreading to Miami-Dade County from areas such as Broward County.

As we began to diagnose cases starting in May 2020, there appeared to be an upward trend for psychosis cases occurring in tandem with COVID diagnoses in our ED. These numbers are shown by the increased COVID-19 cases in Table 2 starting in May. The COVID percentages in May 2020, July 2020, and August 2020 help quantify what part of the total psychosis cases during each given month could be attributed to psychosis and COVID being diagnosed simultaneously. Due to the subjective trend we noted in the beginning, we thought the COVID-19 cases with psychosis would remain elevated and continue to rise higher as the pandemic continued; however, a sharp decrease in June 2020 from 17.17% to 2.50% occurred concerning the number of psychosis admissions in the setting of COVID-19. September and October COVID percentages in 2020 (0%, 4%) also went against this trend (Table 2).

The subsequent decrease in June 2020 and September 2020 was most likely due to hospital bed capacities being met at our facility. At one point, our hospital's medical floor did become saturated and was not accepting COVID patients as admissions from the ED due to a lack of beds. An isolation unit existed for COVID-positive patients in the psychiatric unit; however, capacity was also met here. The entire unit could not be used for COVID due to infection protocols. There was a "COVID wing" to keep uninfected psychiatric patients away from infected psychiatric patients. These events prevented individuals from COVID being admitted to the psychiatry floor. However, we do not have direct data to support this and only have indirect measures such as the length of stay available.

Our data showed that COVID/psychosis patients generally had a more extended stay than patients with psychosis alone (17 days versus five days). The increased length of stay was typically due to them needing a negative COVID test before leaving. So not only did we have limited resources at the time, but those resources were also occupied longer by our COVID/psychosis population, which may have slowed down and inhibited admissions during this time.

Epidemiologically, other possible causes for the decreased incidence of cases at our hospital are clearing the virus and dying from the virus; however, this would allow for a new bed to be open, which contradicts this hypothesis.

During the first wave of COVID-19, the FLDOH reported on July 12, 2020, having had the most cases reported in one day [22]. They also said on July 30, 2020, that COVID-related deaths were at a record high, but not specifically in our area or at our hospital [23].

One would assume that we would have the highest COVID/psychosis cases admitted during this time; however, our data showed results with the highest rate of incidence occurring in August 2020 (Table 2). It is possible that patients died during July, which would have allowed for more resources and admissions in August. Patient death could be why we had our highest COVID with psychosis admission rate in August 2020, but we do not have data to support this claim.

Upper-tailed T-testing showed that the rate of psychosis admissions during our designated "pandemic period" (March 2020 to July 2020) was greater than admissions occurring during the "pre-pandemic period" (October 2019 to February 2020) (p = 0.04053). Two-tailed and lower T-tailed testing was insignificant (Table 8). Since there was a significant increase in the means of psychosis admissions as evidenced by t-testing, we further quantified how large the increase was.

Before the pandemic (10/2019 to 2/2020), there were 90 cases of psychosis (38.8% of 232 total). After the pandemic began (4/2020 to 10/2020), there were 142 cases of psychosis (61.2% of 232 total). Therefore, within our time frame, we witnessed a 22.9% increase in psychosis exacerbation during the pandemic compared to pre-pandemic times. Please note that this does not account for August 2020 to October 2020 (Table 2).

We also attempted to see if COVID could have caused an increase in psychosis admissions using Pearson's correlation coefficient. The relationship between patients having COVID with psychosis and being admitted during this time appeared positive (r = 0.51628); however, not statistically significant (p = 0.06329) (Table 10). As such, we cannot state that COVID-19 had a direct effect on the admissions of psychosis in our ED during the pandemic; however, the increase in psychosis admissions during the pandemic in comparison to before the pandemic was significant.

Our data appear to agree with data showing that psychiatric illness typically becomes exacerbated during epidemics and pandemics [10-12,14-16,19]. However, concerning the increase in psychosis admissions at our facility, we could not establish any specific cause. It should be considered that the trend found for psychosis is not indicative of any single diagnosis, such as schizophrenia, schizoaffective disorder, or unspecified psychosis. Our data only indicate that the patients serviced at our ED presented with more psychotic exacerbations during the COVID pandemic than patients diagnosed with psychotic disorders before the pandemic.

We could not solidify the reasoning for our observed increase in the presentation of psychosis within our population. However, many theories exist attempting to explain the observed trend. These theories describe specifically which psychiatric illnesses became exacerbated and what risk and protective factors exist concerning these psychosis exacerbations occurring during the COVID pandemic.

We could not confidently compare our psychosis data with US prevalence or local data for multiple reasons. First, the annual prevalence data of US citizens reported by the National Alliance on Mental Illness (NAMI) could be used for comparison, but only if our sample population was reflective of the South Miami population.

At first glance, it can be noted that African Americans, Asian Americans, and multi-racial Hispanics appear underrepresented in our data. To this end, we compared our sample demographic data with 2020 US Census Bureau (USCB) data to confirm that our sample population reflected the target population. USCB identified the population of South Miami as having an estimated 11,911 citizens. The most predominant ethnic groups within the population are Caucasian (Hispanic) (40.7%), Caucasian (non-Hispanic) (21.8%), followed by multi-racial (Hispanic) (14.2%), African American (non-Hispanic) (12.9%), and Asian (non-Hispanic) (5.49%) [24]. Our demographics are as follows: Caucasian (Hispanic), Caucasian (non-Hispanic), multi-racial - not represented, African American - not represented, and Asian, not represented. As such, our sample population appears to differ from South Miami's population. However, for the sake of discussion, the assumptions discussed below could be made if our sample population reflected the South Miami population.

As mentioned earlier, a previous psychiatric diagnosis was also found to be a risk factor for hospital admission during the COVID-19 pandemic [19]. The National Association of Counties (NaCO) stated that roughly 9.1% of the population (more than 240,000 individuals) of Miami-Dade County experience serious mental illnesses (SMI; e.g., schizophrenia, bipolar disorder, and major depression) [25]. SMI is considered a more severe subset of mental illnesses; SMI is defined as one or more mental, behavioral, or emotional disorders(s) resulting in severe functional impairment. The Substance Abuse and Mental Health Services Administration (SAMSHA) found that in 2020, the percentage of adults in the US with SMI grew to 5.6%, or 14.2 million people (up from 3.7%, or 8.3 million people, in 2008). In Florida, from 2019 to 2020, SMI increased from 698,168 to 725,329 individuals. Over the same period in Miami-Dade, SMI grew from 92,520 to 93,615 individuals [26,27].

Though no current estimates on SMI in South Miami are available, the burden of psychiatric illness in Miami-Dade County appears higher on average than in the rest of the US. Of note, all our patients had a previous diagnosis of psychiatric illness. The issue has been present for a while too. In 2015, NaCO also stated that less than 13% of patients with psychiatric diagnoses receive care in the public mental health system [25]. It is a known fact that the rate of SMI in Miami-Dade has been increasing over the past 10 years [27]. This fact, coupled with the lack of treatment access, could be another primary reason for the increased trend of psychosis we noticed in our sample.

When comparing our local treatment rates to national treatment rates, South Miami, FL, rates are below the national average. NAMI noted that the annual treatment rate for Hispanics or Latinos ranks second to last compared to other demographic groups nationwide. The rates are as follows: (1) non-Hispanic mixed/multi-racial: 52.2%; (2) non-Hispanic White: 52.4%; (3) non-Hispanic Black or African American: 39.4%; (4) Hispanic or Latino: 36.1%; (5) non-Hispanic Asian: 25.4% [28].

Though the reasons for not receiving treatment are unavailable, 2020 US Census data showed that each year, more and more patients in South Miami identify as not having health insurance. Within the study time frame, the percentage of uninsured citizens in South Miami, FL, grew by 31.9%. Interestingly, 80.8% of the residents in South Miami, FL, were identified as US citizens, much lower than the national average of 93.4%. The trend declined from 2019 to 2020, with the previous rate in Florida being 85.1%. The decrease in US citizenship may cause this population to lack insurance and subsequent access to health care [24]. Though it cannot be a certainty as to why this occurred, the decrease in having US citizenship within the population of South Miami, FL, may serve as a factor.

The FLDOH identified the lack of insurance coverage as an issue in all of Miami-Dade, with 20.7% of the population having no insurance [29]. Unfortunately, the data do not establish which portion of this population has SMI vs. mental illness alone; NAMI estimated that 10.6% of US adults with mental illness had no insurance coverage in 2021, and 11.9% of US adults with SMI had no insurance coverage in 2021 [28]. If the 20.7% uninsurance rate found by FLDOH reflects both groups, then Miami-Dade, on average, would have the same lack of insurance issues that the rest of the US experiences. However, the data lack any information to denote which mental illness population (mental illness vs. SMI) is included in the percentage. FLDOH also identified shortages in mental healthcare providers as another systematic issue. Primary care and dental health professionals are also lacking in Miami-Dade, FL. They did state that their data coincided with data from the sections of the country where low-income residents are concentrated. With patients already at increased risk of decompensation when they have co-occurring and/or co-morbid conditions, it can be assumed that lack of treatment with either or multiple conditions could cause possible hospital admission. FLDOH also cited that resources within the community are available; however, there is often a failure in coordination between providers [29].

It should be noted at this point that lack of access to healthcare can present in multiple manners, including but not limited to lack of insurance, lack of income, and lack of providers, as previously stated. However, it can also occur due to a lack of medication access and the closure of provider locations. Historically psychosis is known to happen with the discontinuation of antipsychotic medication [5]. During the pandemic, not only were there pharmacy closures, but supply chain issues existed, and pharmacy employees were strained. Also, if patients could not get to the pharmacy due to relying on public transportation, they could not refill their medications. Drug Channels Institute stated that the numbers of independent and rural pharmacies went below 20,000 for the first time in 2020 due to closures [30]. Many pharmacists reported being overburdened and/or burnt out during the pandemic. Overall, there was an increase in daily prescription volumes. Combining this with the fact that pharmacists were also looked upon for COVID-19 testing and vaccination, they were often taken away from dispensing medications [31]. Policy changes also limited the number of medicines that could be dispensed [32]. Other causes were due to inadequate personal protective equipment (PPE), lack of resources, and subsequently being exposed to those with COVID-19 [31]. Patients attacked some pharmacy employees due to not receiving their medications [32,33]. Due to fear, anxiety, burnout, and COVID exposure, there were also unexpected absences due to other employees contracting COVID [31]. A survey reported that 8% of physicians' offices closed their offices during COVID due to financial strain. Often the cited reason was steep overheads and reduced patient load [34]. Fear of contracting COVID also kept patients from wanting to visit doctors' offices [35]. Telemedicine was employed; however, many offices could not afford to invest in new technology; it was also reported that it only covered a portion of revenue. Many smaller practices turned to GoFundMe campaigns to help pay the bills [36].

The National Survey on Drug Use and Health (NSDUH) conducted in 2020 stated that for those aged 12 years and older, significant concerns for them during the pandemic were lack of access to medical or mental health care, delayed appointments, not being able to receive prescriptions, and perceived stress from COVID [31].

Those diagnosed with severe mental disorders (SMD), such as psychotic disorders or bipolar disorders, were noted to have more stress and anxiety during lockdown periods when compared to healthy controls. They also displayed poor coping skills, such as increased tobacco smoking, which increased their risk for infection via COVID-19. The disease itself has been associated with a worse prognosis. Though other individuals may be able to develop functional coping strategies to face their anxiety, anxiety could determine unfavorable outcomes in the vulnerable population with SMD, such as triggering a worsening of underlying psychiatric conditions [37]. Further, stress appears to reduce the production of monoamines in the brain, which could also lead to increased anxiety or depression [38]. Psychiatric patients who lived alone during the lockdown and those with a more chronic psychiatric illness were subject to increased distress due to the pandemic. Interestingly, low vitamin D levels seemed to have been associated with distress. Vitamin D protects against chronic stress and inflammation due to hypothalamic-pituitary-adrenal (HPA) axis overdrive [39]. Even after isolation, patients with schizophrenia showed higher stress levels associated with increased C-reactive protein, which also serves as a marker of inflammation [40].

Patients' mental states were also complicated by acute stressors such as changes in relationship status [37]. Social support was identified as an independent variable directly correlated with increased admissions. Individuals that were unmarried, separated, divorced, or otherwise not in a relationship were presented to the ED more frequently for psychiatric evaluations [13]. Fear of COVID-19 due to misinformation also leads to psychological distress in the psychiatric population [41]. Many psychiatric patients also showed increased substance use during this time [19].

It is a well-known fact that certain substances can cause psychosis. Psychosis can be experienced in either the acute intoxication phase, withdrawal phase, or in some cases, with lysergic acid diethylamide (LSD), for instance, after the withdrawal period has subsided. Many classic psychedelic substances such as psilocybin, LSD, mescaline, dimethyltryptamine (DMT), 2C-B, peyote, N-methoxybenzyl, salvia, and ketamine are not regularly tested for upon arrival. Typically only phencyclidine (PCP) and MDMA (3,4-methylenedioxymethamphetamine) are accounted for on a standard 10-panel UDS. Though cocaine and amphetamines could cause psychosis, they usually are classified as stimulants due to their overall excitatory effect. Tetrahydrocannabinol (THC) can act like a hallucinogen and causes psychosis as well. Inhalants such as nitrous oxide (NO) can cause hallucinations and psychosis too. Psychosis could be experienced in withdrawal states, such as alcoholic hallucinosis as well. Many patients could be incorrectly diagnosed with unspecified psychosis if the UDS is negative for any psychoactive substance; therefore, a thorough substance history is critical. Obtaining this history is not always possible in the ED, especially when a patient is acutely psychotic. Furthermore, a positive or negative UDS does not confirm that a substance caused a given condition, especially when false positives exist. UDSs are screening tools, not diagnostic tools. Confirmatory testing does help with this issue, but it is not 100% accurate. In short, using a UDS alone to identify if a substance caused the patient's condition is inadequate. It can aid in the diagnosis, but DSM-V criteria must be utilized to diagnose substance-related psychiatric disorders clinically. Of note, any patient with an acute intoxication or withdrawal was medically stabilized before reaching the psychiatric unit. Regardless, however, we cannot confidently state what relationship substance use has to psychosis exacerbation in our population. Utilization of diagnostic codes for substance use disorders (SUDs) could also introduce bias due to lacking proper specifiers for intoxication or withdrawal symptoms such as acute psychosis. Overall, the primary point of this study was to identify if there was a trend in psychosis admissions during COVID. Secondarily, we tried to associate the increase with only COVID due to available data and, as such, did not account for substance use.

Assuming our sample population matches the target population, we again used NSDUH's 2020 study for comparisons about substance use. The year 2020 marked the first year in which SUDs were evaluated using criteria from the DSM-V, as opposed to criteria from the Diagnostic and Statistical Manual of Mental Disorders, 4th edition (DSM-IV). They further stated that changes were made to the questionnaire too. As such, they advised that care be taken when utilizing their estimates due to actual changes in the population (e.g., the COVID-19 pandemic and other events) from the effects of these methodological changes as such exact numbers were not presented from these data. In quarter 4 of 2020, the majority of individuals aged 12 years and older reported they utilized the same amount of alcohol and drugs before the COVID pandemic started and after the COVID pandemic onset [26].

In our review, low income was also stated to be a risk factor for psychiatric exacerbation. With our hospital being a community hospital, it mainly serves lower-income individuals; however, we do not have data to present to support this. Concerning the employment status and poverty status in South Miami, FL, from 2019 to 2020 was marginally better than national averages. Though many lost their jobs during COVID, the employment rate increased in the South Miami population at 8.72%, from 5.53k employees to 6.01k employees. Poverty rates in South Miami, FL, from 2019 to 2020, are lower than national averages at 12% vs. 12.8%. Regarding homelessness, no local data were available; however, Florida ranks 3rd in the nation concerning numbers of chronically homeless individuals. The rankings are as follows: (1) California - 35,798 individuals, (2) New York - 5,087 individuals, and (3) Florida - 4,915 individuals [24].

A couple of studies showed that those with a positive COVID status typically suffered from poor quality of sleep as well as increased feelings of anxiety and depression [37,42].

In summation, living in poor conditions or being homeless, lacking social support, being unemployed or having no access to a steady income, having low resilience while exposed to increased stress due to COVID-19, having COVID-19 symptoms, having comorbid medical conditions, using substances, lacking access to health services for reasons such as not having insurance, discontinuing medications, getting poor sleep, having generalized inflammation via stress response, lacking mental health providers, and straining of the healthcare system were reported to provoke an exacerbation of psychiatric illness across different populations. Protective factors, therefore, include the following: access to shelter, proper sleep and nutrition, established emotional, financial, and social support systems, uninterrupted access to health care, less perceived stress due to COVID via higher resilience and good coping skills, and having no previous diagnosis or otherwise having stable co-occurring or comorbid conditions.

Any mix of the identified causes in the literature review could have been responsible for the increased trend in psychosis we noted in our population. Each population we looked at had different trends in psychiatric admissions, what diagnoses presented more or less often, and what protective and risk factors were present. Unfortunately, due to a lack of comprehensive data, we can only speculate about conclusions within our population by using generalized data from credible agencies. The state of Florida and governmental agencies have identified an increased burden of mental illness, decreased lack of access to health care, reduced utilization of treatment, and the increased burden of homelessness as significant issues leading to poor outcomes in the mental health services of Miami-Dade. As such, this is our best educated guess as to why we witnessed increased psychosis presentations with our study. Without knowing the socioeconomic status of our sample population, the risk and protective factors outlined are only to be considered as possible associated factors, not causal factors.

Limitations and future considerations

This study was conducted at a single psychiatric emergency service center. Due to this, there are multiple things to consider. Our target population had various ethnicities, such as Asian Americans, African Americans, and multi-racial Hispanics, which were underrepresented in our sample population. Even further, our COVID/psychosis cases were predominately Hispanic and appeared skewed. Therefore, our data do not generally reflect the South Miami, FL, population. It is subjectively reflective of the community immediately surrounding the hospital. Regardless, we were unable to confidently conclude the US population, in general, to quantify further if there was an increase in psychosis in comparison to national averages.

Though an effect was noted in our population, it could be isolated to our local area. However, data still need to be included to assume this. We are not the only hospital in South Miami, FL, that services the community concerning behavioral health. To better understand the distribution of mental illness and SMI in our area, it would be beneficial to do retrospective studies at other hospitals during the same time frame. These studies can then be compiled, reviewed, and analyzed to appreciate the distribution of mental illness in South Miami.

We do not know the distribution of each specific mental illness for each ethnicity in our population or South Miami. To examine this, we would need the prevalence of not only psychosis but also specific International Classification of Diseases, Tenth Revision (ICD-10) diagnoses associated with those admissions from local EDs. Doing this within our time frame could establish a local baseline disorder rates per ethnic group to help draw comparisons as well.

We are also a facility designated by the state of Florida for receiving involuntary admissions for psychiatric evaluations, also known as the Baker Act. This should be considered a possibility for the increased psychosis admissions we documented during our study. Comparisons to other South Miami ED admission rates at institutions with the same designation could also help us understand the trend we noticed.

Much of the information we utilized in the literature review to explain possible causes does not necessarily reflect USA populations or our sample population. Therefore, those proposed causes cannot be assumed to have caused the trend we noted but may further guide our investigation within our study population, especially concerning socioeconomic status. Being a community hospital, the population also included mainly individuals from a lower socioeconomic status; however, we could not quantify this. Designing surveys based on risk factors identified in the literature review would help us better understand how these factors may have contributed to the trend we noticed. To our knowledge, there is no standardized form at this time identifying COVID-related stressors. Including these in future studies would be beneficial. Unfortunately, getting each individual to respond may be challenging because this study is retrospective. We were unable to have follow-up surveys for our population.

Electronic medical record (EMR) data to fill in these gaps is also a possible source of bias due to changes that may or may not be updated, such as employment status, disability status, and marital status EMR data, in general, could be incomplete for multiple reasons besides those mentioned above. EMR and surveys are subject to bias, such as attrition bias, reporting bias, and survey bias, to name a few. Of note, some patients or parents do not consent for their medical records to be used; as such, not all data points for the population are captured. There were also improperly coded diagnoses within our dataset. Table 1 shows chief complaints such as "psych problem" or "age-related cognitive decline" instead of an actual ICD-10 diagnosis like psychosis or dementia. Therefore, forms of bias may exist within our dataset due to the use of EMR data.

The study also only looks at an isolated point of time regarding the entirety of the COVID pandemic and does not consider new variants, strains, or otherwise "phases" of COVID. Also, concerning the time frame from October 2019 to October 2020, we could only use some of our data, which decreased the power of the study. We did not have a year's worth of data from pre-pandemic and pandemic times to compare; we were limited to data from only five months before and after the pandemic was announced. Using the data in this manner could present bias due to issues such as seasonality, which cannot be controlled due to not having corresponding months to limit this. We analyzed as much data as we had hinged on the fact that the WHO made the COVID-19 pandemic official on March 11, 2020. However, we cannot consider seasonality and other factors that could have contributed to psychiatric exacerbation. We attempted to draw comparisons to COVID when factors such as seasonality could not be controlled for. We tried to correlate COVID with the presentation of psychosis to our ED due to study design; it should be noted that this calculation is irrelevant because we could not control for factors that introduce confounding and bias.

Specific data to show when the hospital and psychiatric unit were capped are unavailable. We do not have the data to explain the inconsistencies we believe occurred due to the lack of available hospital beds. We cannot prove our hypotheses of why we saw sharp declines during the pandemic. Data concerning the capacity of hospitals should be considered regarding hospital admissions; this could help us better understand how the patient flow could be managed and help us design a contingency plan for when the hospital is overwhelmed with patient volume.

Concerning our study design, we must note that we grouped all the psychotic disorders in one group, whether the diagnosis was schizoaffective, unspecified psychosis, or schizophrenia. Though our purpose was to identify specifically if psychosis admissions increased, not a specific diagnosis, this could have introduced bias.

Future studies to understand how each diagnosis is affected could help us identify those individuals in our population who are the most vulnerable. We could then allocate resources more effectively and create prevention plans to avoid exacerbating psychiatric illnesses during stressful and trying times.

Utilization of our data outside of South Miami, FL populations should not be attempted, and it should still be carefully used within South Miami until more data can help us understand the distribution of psychiatric illness. According to the FLDOH, data collection is constantly being done. However, they openly stated that a system needs to be in place to integrate data sharing efficiently and thoroughly. Lack of funding for proper health status monitoring was also an issue. Addressing these issues could help us understand the trend we found as well.

## Conclusions

​​The COVID-19 pandemic presented a unique challenge and an opportunity for us to witness how humans adapt to external stressors. Though we cannot prove an exact correlation to any specific stressor, such as COVID, the restrictions put in place, lack of medical resources, or lack of access to available health care, the increase in psychosis during the pandemic was apparent in our dataset. Overall, our population needs more research and data analysis to understand what led to the increase in psychosis truly we noticed in our ED. Systemic issues such as lack of access to health care, lack of providers, and lack of pertinent data such as socioeconomic indicators need to be addressed on the local and state level to understand better why such a sharp increase in psychosis was noted. These data points can help us better understand the distribution of mental illness in our area, which individuals are vulnerable, and how to properly allocate resources for effective intervention and prevention of exacerbations of psychiatric illness.

Overall, this study highlights that patients with chronic disabling diseases such as psychotic disorders are at a higher risk of exacerbation of illness when exposed to external stressors such as a pandemic. It also highlights different ways the healthcare system can be overwhelmed, causing systemic failure, which most often affects vulnerable populations such as those with serious mental illness.
